# Functional electrical stimulation of the facial muscles to improve symptoms in individuals with major depressive disorder: pilot feasibility study

**DOI:** 10.1186/s12938-019-0730-6

**Published:** 2019-11-14

**Authors:** Naaz Kapadia, Vera Zivanovic, Bastien Moineau, Jonathan Downar, Jose Zariffa, Milos R. Popovic

**Affiliations:** 1 Rehabilitation Sciences Institute, University of Toronto, Toronto, ON Canada; 2 Rehabilitation Engineering Laboratory, Lyndhurst Centre, KITE, Toronto Rehabilitation Institute—University Health Network, Toronto, ON Canada; 3 Myant Inc., Toronto, ON Canada; 4 Institute of Biomaterials and Biomedical Engineering, University of Toronto, Toronto, ON Canada; 5 Department of Psychiatry, University of Toronto, Toronto, Canada; 6 MRI Guided rTMS Clinic, Toronto Western Hospital, University Health Network, Toronto, Canada; 7 Edward S. Rogers Sr. Department of Electrical and Computer Engineering, University of Toronto, Toronto, Canada

**Keywords:** Major depressive disorder, Depression, Electrical stimulation, Non-pharmacological, Psychology, Functional electrical stimulation, FES, Functional electrical stimulation therapy, FEST

## Abstract

**Background:**

Currently, the mainstay of treatment in patients diagnosed with major depressive disorder (MDD) requiring medical attention is second generation anti-depressants. However, about 40% of patients treated with second-generation anti-depressants do not respond to initial treatment and approximately 70% do not achieve remission during the first-step treatment. There are a few non-pharmacological options available, but none have shown consistently positive results. There is a need for an intervention that is relatively easy to administer, produces consistently positive results and is associated with minimal side effects. In the current study, we assessed the feasibility of using transcutaneous Functional Electrical Stimulation Therapy (FEST) of the facial muscles, as a tool for improving depressive symptoms in individuals with MDD.

**Results:**

Ten (10) individuals with moderate to severe MDD received three FEST sessions/week for a minimum of 10 to a maximum of 40 sessions. All study participants completed the required 10 therapy sessions, and 5 of the 10 participants completed additional 30 (totalling 40) FEST sessions. There were no adverse events or concerns regarding compliance to therapy. We found statistically significant improvements on Hamilton Rating Scale for Depression (HDS) and Inventory of Depressive Symptomatology (IDS) measures. However, no significant improvements were found on Positive and Negative Affect Scale and 10-point Visual Analogue Scale scales. Participants reported improvements in sleeping patterns, and this correlated with statistically significant improvements on sleep parameters of HDS and IDS measures.

**Conclusion:**

This study indicates that facial FEST is an acceptable, practical, and safe treatment in individuals with MDD. We provide preliminary evidence to show improvements in depressive symptoms following a minimum of 10 FEST sessions.

## Background

Major depressive disorder (MDD) is defined as the presence of depressed mood or loss of interest or pleasure, along with at least 4 additional MDD diagnosis criteria or symptoms (weight gain, weight loss, insomnia, psychomotor retardation, fatigue, feelings of worthlessness, diminished ability to think/concentrate, suicidal ideation/attempt) for at least 2 weeks [1]. MDD is the most prevalent and disabling form of depression, affecting more than 16% of U.S. adults in their lifetime and in any given year at least 7% of adult population experience an MDD episode requiring medical attention. In Canada, the overall annual prevalence of major depressive episode is 4.7% and the lifetime prevalence is 11.3% [1]. MDD is more common in females (male:female ratio is 1:2), in younger people, in singles (never married), in previously married people who are divorced or separated, in those who have one or more chronic medical conditions, and in those unemployed within the past year [2]. According to the World Health Organization, by 2020 depression will cause more disability than infectious diseases, cancer, or accidents, and it will be second only to ischemic heart disease as a cause of disability [3]. Clinically, MDD is characterized as mild, moderate, or severe based on symptom severity, functional impairment, and level of patient distress.

The etiopathogenesis of MDD is based upon multiple factors that may act at different levels (biological, genetic, psychological, and social). Mechanisms involved in the emergence of depressive episodes are multifactorial and not yet fully understood [4]. The mainstay of treatment in patients diagnosed with MDD requiring medical attention is second generation anti-depressants [5]. However, about 40% of patients treated with second-generation anti-depressants do not respond to initial treatment and approximately 70% do not achieve remission during the first-step treatment [6]. Non-pharmacological interventions for MDD include cognitive behavioural therapy, complementary and alternative medicine options, and exercise [7]. Amongst these, cognitive behavioural therapy has shown the most promising results, comparable to those of second-generation anti-depressants [7].

Given the low response rate of second-generation anti-depressants and the high incidence of MDD, there is a need to develop and test new interventions that are easy to administer, have less side effects and high response rate. To this effect, some newer interventions that are currently being researched include repeated transcranial magnetic stimulation (rTMS) [8, 9] and transcranial direct current stimulation (tDCS) [10, 11]. There is some evidence regarding the effectiveness of 20–40 min sessions of rTMS delivered 5 days a week for 4 to 8 weeks in patients with drug resistant MDD [12]. However, this treatment must be administered in a clinical setting and cannot easily be adapted for home use, which makes access to therapy somewhat difficult for patients. The exact mechanism of action of rTMS remains.

to be elucidated, however, there is evidence that dopaminergic neurotransmission is involved, at least for rTMS over prefrontal and motor areas. Such evidence comes from animal models [13] as well as human studies [14, 15]. These studies have shown increased dopamine transmission not only in subcortical areas but also in medial prefrontal areas, after TMS. The clinical utility of tDCS as a treatment for MDD remains unclear when clinically relevant outcomes such as response and remission rates are considered [1].

The current study aimed to explore transcutaneous functional electrical stimulation therapy (FEST) of facial muscles as a potential treatment for MDD. A substantial body of research has been devoted to the study of facial movements as they relate to particular emotions. Facial expressions for basic emotions (happiness, fear, surprise, etc.) have been found to be well-defined and universal across cultures [16]. Certain facial muscle movements can be easily controlled voluntarily, while others occur primarily during “genuine” emotions. For example, voluntary smiles (e.g. for social purposes, without any particular emotional involvement) usually consist only of the upward curving of the lips, whereas spontaneous smiles due to positive emotions also involve the eyes (the so-called “Duchenne marker”), characterized by a rising of the cheeks and the appearance of crows-feet wrinkles next to the eyes [17, 18]. These two facial expressions are mediated by different neural pathways: (i) voluntary smiles are initiated in the motor cortex and routed via the pyramidal motor system, whereas (ii) involuntary smiles arise mainly from subcortical nuclei and are routed via the extrapyramidal motor system. An unexpected by-product of this research has been the observation that voluntarily producing and holding an expression can induce the corresponding emotion [19, 20]. This effect is more pronounced when a person pays specific attention to voluntarily activating muscles that are usually only used involuntarily (e.g. the Duchenne marker) [21, 22].

Functional electrical stimulation is a technique in which muscles are electrically stimulated, causing them to contract. Functional electrical stimulation has been shown to have therapeutic applications: artificially stimulating paralyzed or weakened muscles while the individual attempts to voluntarily contract those same muscles can lead to significant functional improvement in ability to voluntarily control previously paralysed muscles [23]. This use of functional electrical stimulation is called functional electrical stimulation therapy (FEST). Recent studies have shown that this process is accompanied by plasticity in the central nervous system, with regions affected by the injury and associated with the stimulated muscles displaying increased activity [24].

The FES protocol of the zygomatic major and orbicularis oculi is rooted in the Facial Feedback Theory. The idea that afferent feedback from expressive behavior may play a causal role in the experience of emotion takes its roots in part from Charles Darwin’s and William James’s views [25, 26]. The influence of these views can be seen in more recent theories of emotion, which assign to facial expression a primary role in the subjective experience of emotion and this gave rise to the facial feedback hypothesis [27, 28], which states that facial movement could influence emotional experience [29]. These results have been strongly supported in botulinum toxin studies where botulinum toxin of the corrugator muscle has resulted in decreased activation of brain regions implicated in emotional processing and emotional experience (namely, the amygdala and the brainstem) [30]. In this study, we choose to stimulate muscles responsible for “genuine smile” rather than depressing the activity of the corrugator muscle.

In individuals with MDD alterations in the neural circuitry and neurotransmitters of the prefrontal cortex and the limbic system are implicated. Functional MRI studies suggest a decrement in the “communication” between amygdala and anterior cingulate cortex regions, explaining a decreased capacity of the cortex to regulate subcortical areas mediating negative emotions, usually referred to as a deficit in “top-down” control [31]. Moreover, magnetic resonance images (MRI) of the brains of patients with depression have identified differences in both structure and function compared to non-depressed subjects. Although some inconsistency exists, meta-analysis has confirmed smaller hippocampal volumes [32]. The loss of hippocampal neurons correlates with impaired memory and dysthymic mood. We hypothesize that FES of the facial muscles will result in up-regulation of neuronal activity in the limbic system that will result in improved mood regulation.

Our primary objective was to explore the feasibility of applying FEST to the facial muscles involved in involuntary smile and to obtain preliminary evidence regarding the effect of FEST on symptoms related to MDD. We hypothesized that applying FEST to the facial muscles associated with smiling (including the Duchenne marker) is feasible and safe and that it might help augment the symptoms of MDD.

## Results

Ten participants that fit the inclusion/exclusion criteria were recruited. At the time of admission, as rated by IDS, 1 participant had very severe depression, 3 had severe depression and 6 had moderate depression. All participants completed 10 sessions of FEST within the allocated time, and 5 participants continued with FEST until they completed 40 sessions (i.e. received 30 additional sessions). No adverse events were reported.

All 10 participants showed improvement in their depressive symptomology as measured by IDS and HDS upon completion of 10 sessions of FEST, and 4 out of the 5 participants continued to show further improvements upon completion of 40 sessions (Table 1).

**Table 1:** Participant raw scores on Quantitative study outcome measures

Outcome measure	IDS			HDS			VAS			PANAS					
Participant ID	Pre	10 sess.	40 sess.	Pre	10 sess.	40 sess.	Pre	10 sess.	40 sess.	GNA Pre	GNA 10 sess.	GNA 40 sess.	GPA Pre	GPA 10 sess.	GPA 40 sess.
AANM	41	30		23	14		70	70		30	20		11	13	
AANN	25	18		9	6		50	50		16	11		10	14	
AANO	26	11		16	4		30	50		30	20		15	14	
AANS	50	13	11	24	4	3	70	45	45	26	14	10	21	14	20
AANT	37	14	10	21	13	3	85	65	40	19	12	11	15	13	20
AANU	27	13		13	3		50	50		28	30	26	26	24	26
AANZ	28	15	12	14	7	4	25	17	50	11	11		22	18	
AANW	44	24	36	16	7	9	70	65	65	35	27	41	24	35	33
AANY	29	28		12	11		55	55		14	10		23	26	
AANX	43	44	43	14	12	9	60	35	57	21	13	10	26	34	25

Pre-baseline score; 10 sess. - after 10 therapy sessions; 40 sess. -after 40 therapy sessions

*IDS* Inventory of Depressive Symptomatology, *HDS* Hamilton Rating Scale for Depression, *VAS* Visual Analog Scale, *PANAS* Positive and Negative Affect Scale, *Pre* baseline, *Post* after 10 FEST sessions, *GNA* general negative affect; *GPA* general positive affect

**Fig. 1:**
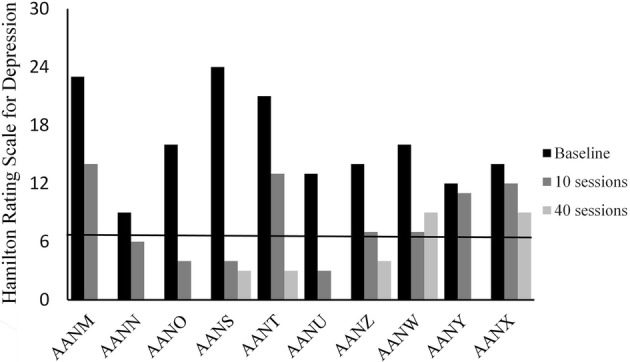
Hamilton Rating Scale for Depression (HDS) scores at baseline and upon completion of 10 sessions of the Functional Electrical Stimulation Therapy (FEST) of the facial muscles (values between 0 and 6 indicate no depression). *X*-axis shows the 10 participants of the study

**Fig. 2:**
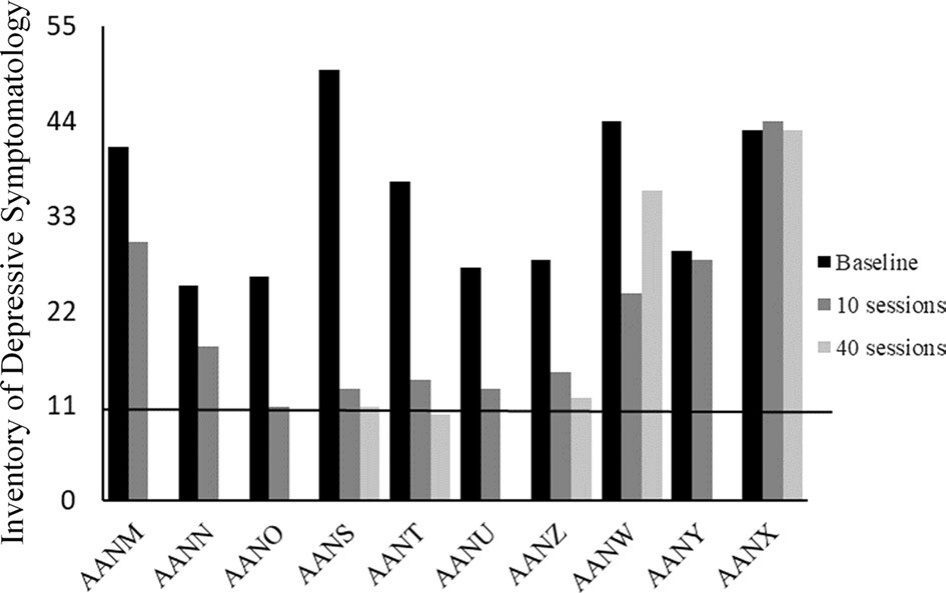
Inventory of Depressive Symptomatology (IDS) scores at baseline and upon completion of 10 sessions of the Functional Electrical Stimulation Therapy (FEST) of the facial muscles (values between 0 and 11 indicate no depression). *X*-axis shows the 10 participants of the study

We found statistically significant improvements on both IDS and HDS upon completion of 10 FEST sessions (Figs. 1, 2). The average total score on IDS was 35 at baseline and 21 upon completion of 10 FEST sessions (Mean change score of 14, SD = 11.05, *p* = 0.008). Similarly, the average total score on HDS was 16.2 at baseline and 8.1 upon completion of 10 sessions (Mean change score of 8.1, SD = 5.26, *p* = 0.005).

No statistically significant changes were seen on the VAS. The average score on VAS was 53.5 at baseline and 50.2 upon completion of 10 sessions (Mean change score of 3.3, SD = 16.76, *p* = 0.481). On the PANAS there were statistically significant improvements on general negative affect (Mean change score of 6.2, SD = 4.49, *p* = 0.011), sadness (Mean change score of 3.4, SD = 3.50, *p* = 0.011), guilt (Mean change score of 4.5, SD = 4.08, *p* = 0.012) and hostility (Mean change score of 2.7, SD = 4.11, *p* = 0.05), whereas none of the domains under positive affect reached statistical significance.

### Secondary exploratory analyses

**Fig. 3:**
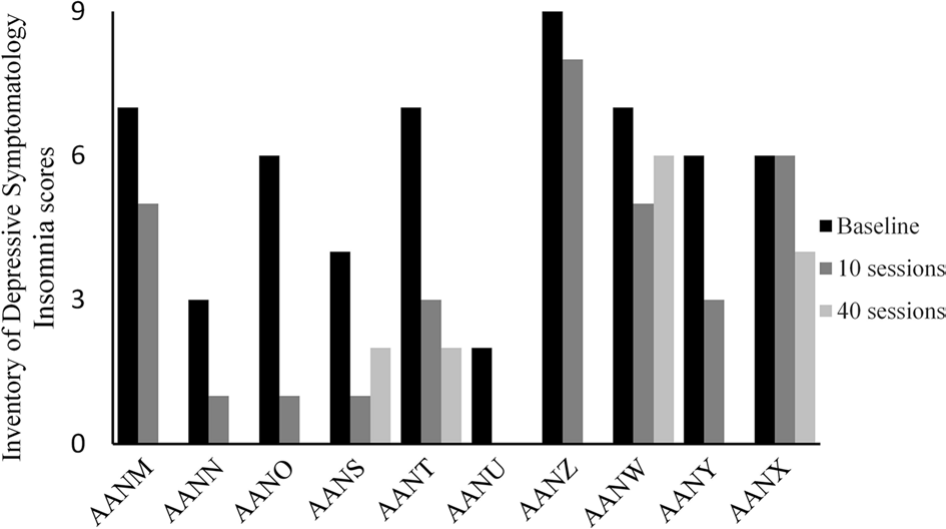
Inventory of Depressive Symptomatology (IDS) insomnia scores at baseline and upon completion of 10 sessions of the Functional Electrical Stimulation Therapy (FEST) of the facial muscles (lower values indicate better sleep with 0 indicating normal sleep patterns.). *X*-axis shows the 10 participants of the study

Many of the participants described an improvement in their sleep pattern (see Interview results below). Although IDS and HDS scores are valid only as total, we analyzed specifically the sleep parameters on IDS and HDS, at baseline and after 10 sessions. On the insomnia parameters of the IDS we found a statistically significant improvement on sleep onset insomnia (Mean change score of 1, SD = 0.81, *p* = 0.015) (Fig. 3) and mid nocturnal insomnia (Mean change score of 0.9, SD = 0.99, *p* = 0.034), whereas the scores approached statistical significance for early morning insomnia (Mean change score of 0.5, SD = 0.70, *p* = 0.059). Similarly, on HDS the scores on insomnia early (Mean change score of 0.7, SD = 0.67, *p* = 0.020) and insomnia middle (Mean change score of 0.6, SD = 0.51, *p* = 0.014) reached statistical significance.

### Qualitative data

A total of 13 interviews were conducted: 9 interviews after 10 sessions, and 4 after 40 sessions of FEST.

#### Close-ended questionnaire

Responses to the final questionnaire conducted are reported in Table 2.

**Table 2:** Results from the close-ended questionnaires after the participants’ last session (10th or 40th session)

Question	How pleasant or unpleasant did you find the FES sessions to be?				
Answers	Very unpleasant	Unpleasant	Neither pleasant nor unpleasant	Pleasant	Very pleasant
Results (*n*)	1	0	2	4	3
Question	Do you feel confident that this treatment could result in an improvement in symptoms of depression?				
Answers	Not at all confident	Not confident	Neither confident nor unconfident	Confident	Very confident
Results (*n*)	0	1	2	4	3
Question	If the FEST treatment were found to result in a mild improvement to symptoms of depression, how likely would you be to choose to undergo this treatment in the future?				
Answers	Very unlikely	Unlikely	Neither likely nor unlikely	Likely	Very likely
Results (*n*)	0	0	0	5	5
Question	If the FEST treatment were found to result in a moderate improvement to symptoms of depression, how likely would you be to choose to undergo this treatment in the future?				
Answers	Very unlikely	Unlikely	Neither likely nor unlikely	Likely	Very likely
Results (*n*)	0	0	1	2	7
Question	If the FES treatment were found to result in a strong improvement to symptoms of depression, how likely would you be to choose to undergo this treatment in the future?				
Answers	Very unlikely	Unlikely	Neither likely nor unlikely	Likely	Very likely
Results (*n*)	0	0	0	2	8

Seventy percent of the participants found the intervention pleasant and 70% were confident that it could improve symptoms related to depression. All of them reported that they would likely or very likely choose to undergo this treatment again. There were only two close-ended answers with a negative connotation toward the intervention. Participant **AANS** reported the intervention as “very unpleasant”, but said in the interview he liked “*everything*” about the sessions and described FEST sessions as “*comfortable*”, to the exception of the “*sticky stuff on the face*” (the gel electrodes). Participant **AANW** reported, being “not confident” that this treatment could result in an improvement in symptoms of depression, but reported having reduced frequency, duration and severity of anxiety and depression events.

#### Structured interview (open-ended questions)

The final coding framework contained 16 codes, unequally used, which were divided in 3 themes: Intervention Effects, Trial Execution, and Participants’ Suggestions (Table 3).

**Table 3:** Coding framework and results for the open-ended questions in the 13 interviews (after 10th and/or 40th session)

Name of the theme	Intervention effects	Trial execution	Participants suggestions
Code 1	Relaxation-effect-immediate (*n* = 9)	Satisfaction-procedure (*n* = 9)	Electrodes-issue (*n* = 3)
Code 2	Mood-improvement (*n* = 8)	Transportation-issue (*n* = 5)	Practical-considerations (*n* = 3)
Code 3	Sleep-improvement (*n* = 7)	Scheduling-issues (*n* = 3)	Insufficient-number-session (*n* = 2)
Code 4	Sleepy-effect-immediate (*n* = 3)	Life-circumstances (*n* = 2)	Home-use-device (*n* = 1)
Code 5	Energy-improvement (*n* = 2)		Desire-people-interaction (*n* = 1)
Code 6	Appetite-improvement (*n* = 1)		Smile-purposely (*n* = 1)

*n* is the number of participants whose responses were coded at least once with the code, out of 10 participants

#### Intervention effect

Participants reported effect of the FEST sessions on relaxation, mood, sleep, energy, and appetite. Some effects were felt immediately following the session: feeling relaxed (“*After session I felt good, relaxed*” **AANN**; “*It is slowing me down and relaxing, it is positive experience*” **AANY**) and/or feeling sleepy (“*Feeling sleepy afterward. […] When I come home I have to have nap*” **AANX**; “*After session I have desire to rest, to sleep*” **AANT**). Other effects were described as more general (and not just immediate): mood improvement (“*Ongoing depression and anxiety but events are shortened. Duration is down, frequency is down, and amplitude is down, going back to normal confident quicker*” **AANW**; “*I am able to tolerate things. […] I felt better*” **AANU**; “*I noticed that I am more happy than usual*” **AANU**; “*Mood is better, did not cry as I did before*” **AANS**) and sleep improvement (“*I slept yesterday night and last several days 5*–*6* *h per night, which is very rare otherwise*” **AANY**; “*After 5th session I slept for 5* *h, this does not happened before. I usually wake up every 2* *h*” **AANW**; “*My sleep is better, easier to concentrate. […] When I do not sleep well, I cannot function. But this increase in sleep helps me function better*” **AANU**). Two participants reported energy improvement (“*I felt a little bit better and my energy level improved*” **AANZ**), and 1 reported appetite improvement (“*My appetite increase*” **AANU**).

#### Trial execution

Nearly all participants reported satisfaction with the procedure (“*I felt better while I am here and also thankful that somebody is trying to find cure for depression*” **AANM**; “*No side effects. It is perfect*” **AANO**), however, voiced personal difficulties related to transportation (“C*oming is hard because I live far away*” **AANM**), scheduling (“*40 sessions is too much for coming*” **AANX**), or life circumstances (“*Things are difficult in my life, I have problem with work*” **AANN**).

#### Participants suggestions

Participants identified opportunities for improvement related to electrodes application (“*Is there any easier way to put electrodes? They keep falling off unless they are glued*” **AANY**), delivery of therapy (“*Maybe if participants have headphone so that I do not disturb others*” **AANW**), and number of sessions (“*More sessions would be more beneficial*” **AANN**).

A few participants suggested solutions inspired from their participation in the study: one suggested to “*Create machine for home use*” (**AANT**) to allow immediate rest, and to avoid transportation hassle. Another reported that she actively pursued the artificial smile approached on her own (“*I do not feel change in mood. I smile more on purpose*” **AANO**); and another expressed the desire for more human interactions (“*I think people with mental health need more interaction with people*” **AANU**).

In the above reporting, results after 10 and 40 sessions were gathered (13 interviews total). All four participants who did interviews after 40 sessions expressed satisfaction with the procedure, mood and sleep improvement, and an immediate relaxation effect.

## Discussion

The burden attributed to MDD remains high, whether from individual distress, functional and relationship impairment reduced quality of life, or societal economic cost [1]. Hence, as the societal burden and financial costs of MDD continue to escalate, so does the need for evidence-based and cost-effective interventions that demonstrate improvement in functioning [33]. Given the comparable results of non-pharmacological and pharmacological interventions and the higher rate of adverse events as well as treatment drop-outs with second-generation anti-depressants, researchers and clinicians are working towards developing therapies with higher response and compliance rates and fewer side effects. To this end, several neurostimulation treatments are being investigated: rTMS, tDCS, magnetic seizure therapy (MST) and vagus nerve stimulation (VNS). Amongst all of these rTMS has shown the most promising results. However, rTMS can be delivered only in a controlled setting and requires the presence of a medical doctor, which limits its implementation. Also, in the absence of maintenance doses, rTMS has a high incidence of relapse, with an onset as early as 24 weeks post-treatment [1]. On the other hand, research related to tDCS has shown very small effect sizes bringing into question the clinical and functional benefits of this therapy. Besides, there are no studies examining safety and tolerability of tDCS over long-term use. The other two neurostimulation techniques are still considered investigational. Thus, there remains an outstanding need for an intervention that is safe, well tolerated, easy to administer, and one that has high response-low remission rates.

In the current study, we aimed to address the gap by assessing the feasibility of delivering transcutaneous facial FEST, in individuals with MDD. More specifically, we aimed to look at the acceptability of the intervention, the practicality of its application, and a limited efficacy testing. We hypothesized that the treatment is feasible and will result in improvement in symptoms of MDD. To the best of our knowledge, this is the first study looking at this type of intervention.

From an acceptability perspective, the goal was twofold. Firstly, we assessed if the intervention was well tolerated by the intended patient population and secondly if it was feasible to be delivered by allied health professionals without direct supervision of a medical doctor. The results of the study demonstrated that the intervention is safe and well tolerated by study participants. All study participants completed the minimum 10 FEST sessions and the qualitative data revealed that 7 of the 10 participants found the therapy “pleasant” or “very pleasant”. From an application perspective, the therapy was easy to administer, and donning and doffing time was approximately 15 min. No adverse events were reported over the course of therapy and hence the intervention could be delivered without immediate medical supervision.

From a practicality perspective, the focus was to explore the need for resources, time and commitment both on part of study participants as well as the research team. Originally, the study was planned to deliver 10 FEST sessions in 2 weeks period (1-h session per day/5 days a week). However, our study participants reported that it was not feasible for them to come 5 times per week and so we modified the protocol to reduce the therapy sessions to 3 days per week. All study participants were able to successfully follow this regime. The study participants as well as the research team found that a 1-h commitment per session was suitable. The setup and administration of therapy required no more than one person. Thus, from a practicality perspective the adjusted intervention frequency and intensity could be easily adapted in a clinical setting without excessive stress on resources in terms of both time and personnel.

Further, the results provide preliminary evidence to show that stimulating muscles involved in producing a Duchenne smile may have a potential to improve depressive symptoms in individuals with MDD. In the current study, the participants showed an average 50% decrease in scores on the HDS upon completion of 10 sessions, which is considered as a positive response to therapy. Moreover, six of the ten participants scored less than or equal to 7 post-treatment, which is indicative of them being in remission. Similarly, there was a statistically significant reduction in scores on the IDS after 10 sessions of FEST.

The theme of “improvement in sleep” emerged as an important finding from the Qualitative data. To explore this further, we analyzed the insomnia parameters on both IDS and HDS separately and found statistically significant improvements in early and midnight insomnia on both IDS and HDS measures. This is a very important finding given that insomnia and sleep complaints are prominent symptoms of depression, and are shown to demonstrate an increased risk of coronary artery disease, morbidity and death [34], as well as increased suffering and functional impairment in MDD. Effective, evidence-based treatments for insomnia in MDD are an unmet need in clinical practice [35]. Facial FES might have the potential to target depressive symptoms, including insomnia.

The current study did not show any significant improvement on the PANAS Positive Affect (PA) scores, but there were statistically significant improvements on the general Negative Affect (NA) score (“sadness”, “guilt” and “hostility”). In light of the results on HDS and IDS, the changes on PANAS might seem counterintuitive. However, there is evidence in the literature to show that affect changes may progress through a course of decrease in Negative Affect followed by an increase in Positive Affect [36]. Also given the experimental nature of our treatment there is a likelihood that the central nervous system pathways engaged may be better suited to suppress negative emotions. Reductions in Negative Affect can lead to increases in Positive Affect through several pathways. First, individuals who have experienced reductions in Negative Affect may begin to feel more hopeful about the future due to their improvement, which results in an increase of Positive Affect. Second, high and chronic levels of Negative Affect have been found to result in interpersonal problems and rejection [37]. Therefore, reductions in Negative Affect may enhance depressed individuals’ ability to seek and receive social support, which in turn could enhance Positive Affect. It is important to note that these pathways remain speculative and future research is required to examine them directly [36].

Despite the positive results of the study, there are some limitations which should be taken into consideration while interpreting the study results. Although our study showed that facial FEST is a safe and well-tolerated treatment option with 100% adherence rate, the efficacy results of the study are very preliminary in nature. The results related to treatment effectiveness should be interpreted with caution due to the lack of control group and high rate of placebo effect in individuals with depression. The lack of follow-up assessment did not allow us to explore the “relapse” rates.

## Conclusion

This is the first study exploring the use of Functional Electrical Stimulation Therapy (FEST) as a possible treatment for patients with major depressive disorder. The findings of the study indicate that transcutaneous FEST of the facial muscles is a safe and viable treatment for MDD. The study provides preliminary evidence that facial FEST therapy results in an improvement in mood and an improvement in sleep patterns in individuals with MDD.

## Methods

The current study was a mixed-method pilot interventional study conducted at a Rehabilitation Hospital. The aim of this study was to explore the feasibility of applying FEST to the facial muscles involved in involuntary smile in individuals with MDD. The secondary aim was to obtain preliminary evidence regarding the effect of FEST on symptoms related to MDD.

### Participants

A convenience sample of 10 individuals with clinically diagnosed MDD and who met the inclusion/exclusion criteria was recruited to the study. Participant demographics are presented in Table 4.

**Table 4:** Participant Demographics

Patient ID	Age	Sex	Antidepressant medication	rTMS (time prior to start of study intervention)	Range of stimulation amplitude in mA
AANM	42	F	No	Yes (1.5 months)	9–12
AANN	43	M	Yes	Yes (4 months)	11
AANO	58	M	No	No	9–12
AANS	52	F	Yes	No	8–11
AANT	57	F	No	No	8–12
AANU	34	M	No	No	12
AANZ	45	M	Yes	No	9–10
AANW	62	M	No	Yes (6 months)	11–13
AANY	65	M	No	No	11–13
AANX	60	F	No	No	7–9

*rTMS* transcranial magnetic stimulation

### Procedure

Appropriate Ethics Board approval was obtained, and participants were recruited through posters posted at our hospital. Participants reviewed the informed consent forms with the research coordinator and once all participant questions were answered then consenting participants signed the informed consent forms.

The inclusion criteria were

1. No change in the medication regimen or other forms of treatments for at least 4 weeks prior to beginning the study. This was established through self-report, in combination with an Antidepressant Treatment History Form, which was filled out by the participant themselves or with the assistance of their pharmacist.
2. Moderate to very severe depression as indicated by a score between 25 and 50 on the Inventory of Depressive Symptomatology scale; and.
3. Age between 18 and 70 years.

The exclusion criteria were

1. History of epilepsy or seizures.
2. History of damage or dysfunction to facial nerves.
3. Individuals with metal orthopedic implants in the mouth (e.g. plates or screws).
4. Individuals suffering from fibromyalgia.
5. Individuals currently receiving any form of transcranial brain stimulation [e.g. rTMS, Electro Convulsive Therapy (ECT), or Magnetic Stimulation Therapy (MST)]
6. Individuals with past or current symptoms of mania, hypomania, mixed episodes, psychotic disorders, obsessive–compulsive disorder, or post-traumatic stress disorder.
7. Individuals with current substance abuse or dependence.
8. Individuals with current suicidal intent or plan.
9. Individuals not able to understand instructions in English; and
10. Individuals unable to produce the required expression with FEST (“Duchenne marker”).

### Intervention

**Fig. 4:**
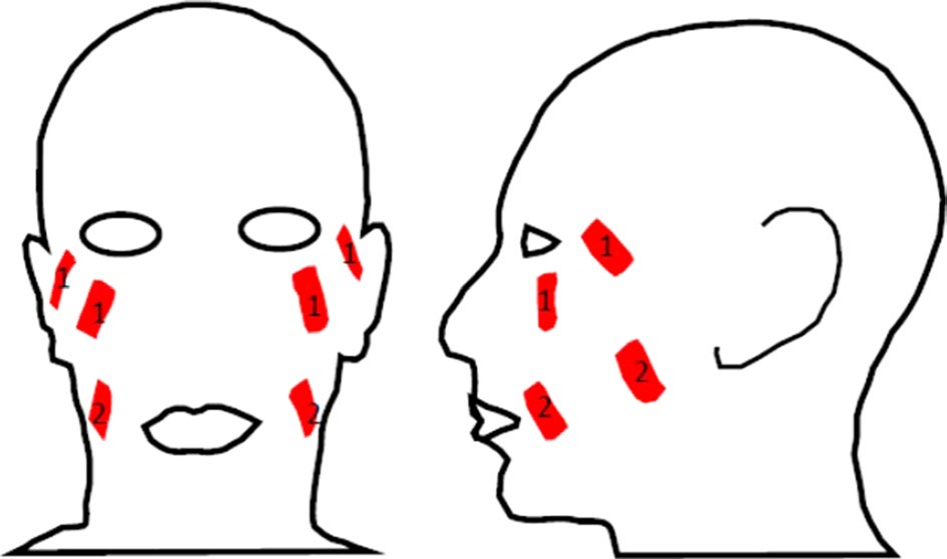
Electrode placement for the orbicularis oculi and the zygomatic major muscles (Electrode 1-Orbicularis Oculi and Electrode 2-Zygomatic Major)

All participants recruited to the study received facial FEST 3 times per week for a minimum of 10 sessions, with an option to continue up to 40 sessions. The FEST sessions were administered by either a Research Associate who is a Physician by training (not licensed to practice in Canada) or a licensed Physiotherapist. Each FEST session lasted 1 h, which included donning and doffing the electrodes (~ 15 min) and consisted of alternating periods of stimulation and rest, 15 s each. FEST was delivered using Compex Motion stimulator (Compex SA, Switzerland). Surface adhesive electrodes were placed bilaterally on the zygomatic major and orbicularis oculi muscles (Fig. 4). FEST parameters for stimulation used were 150 μs biphasic pulses delivered at 60 Hz, with amplitudes in the range of 1–15 mA [38]. For individual participant amplitude range please refer to Table 4. Note that the zygomatic major and orbicularis oculi are the two muscles required to produce an expression of happiness according to the Facial Action Coding System (FACS, [39]), though only the orbicularis oculi is specific to genuine smiles [17, 19]. During the periods of FEST, the participants were required to attempt to voluntary produce the desired expression (smile with the Duchenne marker). Participants were shown comedy videos throughout the sessions, firstly as a way to make participation in the study more pleasant and ensure compliance, and secondly because this assisted the participants to produce more genuine smiles. Participants were given an option to select their own videos from a collection of videos on Netflix.

### Quantitative outcome assessments

The feasibility of the study was determined based on participant’s compliance with treatment and the incidence of adverse events. The following assessments were used to identify change in MDD symptomatology and were administered at baseline, upon completion of 5 sessions, 10 sessions and 40 sessions (for those who choose to do 40 sessions):

- Inventory of Depressive Symptomatology (IDS): The IDS is a 30-item questionnaire that assesses all the criterion symptom domains designated by the American Psychiatry Association Diagnostic and Statistical Manual of Mental Disorders—4th edition [40] and the 5th edition to diagnose a major depressive episode. Higher scores indicate increased severity of depression [41, 42].
- Hamilton Rating Scale for Depression (HDS): The HDS is a 21-item multidimensional and clinician-rated scale that has become the standard for clinical trials of depression. For the 17-item version of the HDS, scores can range from 0 to 54. It is generally accepted that scores between 0 and 6 do not indicate the presence of depression, scores between 7 and 17 indicate mild depression, scores between 18 and 24 indicate moderate depression and scores over 24 indicate severe depression [43, 44]. A decrease in scores of at least 50% typically indicates response, and scores of 7 or less after treatment indicate remission.
- Positive and Negative Affect Scale (PANAS-X): The PANAS-X comprises two mood scales, one that measures positive affect (PA) and one that measures negative affect (NA). Ten descriptors are used for each affect to define their meanings. Participants were required to respond to a 60-item test using 5 point scale that ranged from very slightly or not at all (1) to extremely (5) [45].
- A 10-point Visual Analogue Scale (VAS) for mood: A VAS ranging from 0 to 100, with intervals of 10, was used. Zero on the scale indicated no depressive symptoms and 100 indicated severe depression.

### Statistical analysis

Descriptive statistics was used to present participant demographics. Baseline scores and scores upon completion of 10 FEST sessions on the HDS, IDS, PANAS and VAS were compared using non-parametric Wilcoxon Signed Rank Test. A *p* < 0.05 was considered statistically significant. Since only 5 of the 10 participants completed 40 FEST sessions, statistical analysis was not performed on outcomes collected after the 40th session. Instead these results are reported in a descriptive format.

### Qualitative outcome assessments

After the 10th session, and 40th session for those who completed 40 sessions, a structured interview was conducted by the research coordinator who delivered the FEST sessions (VZ).

The participants were asked close-ended questions pertaining to “how pleasant were the FEST sessions”, “how confident they were about effect on depression symptoms”, and “how likely they would be to undergo this treatment in the future” (Table 3). A short structured interview was conducted using open-ended questions to identify the positive (“What did you like about the FEST sessions?”) and negative (“What did you not like about the FEST sessions?”) aspects of the FEST, as well as to gather participant suggestions for improvement. Participant’s answers were noted by the investigator on a paper form, and latter transcribed electronically.

Fundamental qualitative description methodology [46] was employed for the content analysis of the transcripts. This was meant to limit the interpretation of the content and ensure the descriptive validity of the analysis. Iterative investigator triangulation (separate coding, in parallel, followed by comparison and discussion) was used at each stage of the analysis process to ensure the trustworthiness of the data [47]. An open coding process was used initially: two researchers (BM and VZ) independently read each transcript and assigned codes to text selections that contained information relevant to the study. Then, they designed, by consensus, a coding framework of 16 codes that was then re-applied to the transcript in a second round of separate coding, after which they reached agreement. The final step involved the identification of themes, defined as a recurring category or connection made between categories [47]. Themes were developed separately first, and then a final theme framework was defined by consensus between BM and VZ.

## Data Availability

The datasets used and/or analysed during the current study are available from the corresponding author on reasonable request

## Abbreviations

MDD: major depressive disorder
FEST: functional electrical stimulation therapy
HDS: Hamilton Rating Scale for Depression
IDS: Inventory of Depressive Symptomatology
FES: functional electrical stimulation
rTMS: repeated transcranial magnetic stimulation
tDCS: transcranial direct current stimulation
TMS: transcranial magnetic stimulation
MRI: magnetic resonance imaging
VAS: Visual Analog Scale
PANAS: Positive and Negative Affect Scale
SD: standard deviation
MST: magnetic seizure therapy
VNS: vagus nerve stimulation
PA: positive affect
NA: negative affect
ECT: Electro Convulsive Therapy
FACS: Facial Action Coding System
